# A Comparison of Blue Light and Caffeine Effects on Cognitive Function and Alertness in Humans

**DOI:** 10.1371/journal.pone.0076707

**Published:** 2013-10-07

**Authors:** C. Martyn Beaven, Johan Ekström

**Affiliations:** 1 Swedish Winter Sports Research Centre, Department of Health Sciences, Mid Sweden University, Östersund, Sweden; 2 Department of Psychology, Mid Sweden University, Östersund, Sweden; MRC - NIMR, United Kingdom

## Abstract

The alerting effects of both caffeine and short wavelength (blue) light have been consistently reported. The ability of blue light to enhance alertness and cognitive function via non-image forming neuropathways have been suggested as a non-pharmacological countermeasure for drowsiness across a range of occupational settings. Here we compare and contrast the alerting and psychomotor effects of 240 mg of caffeine and a 1-h dose of ~40 lx blue light in a non-athletic population. Twenty-one healthy subjects performed a computer-based psychomotor vigilance test before and after each of four randomly assigned trial conditions performed on different days: white light/placebo; white light/240 mg caffeine; blue light/placebo; blue light/240 mg caffeine. The Karolinska Sleepiness Scale was used to assess subjective measures of alertness. Both the caffeine only and blue light only conditions enhanced accuracy in a visual reaction test requiring a decision and an additive effect was observed with respect to the fastest reaction times. However, in a test of executive function, where a distraction was included, caffeine exerted a negative effect on accuracy. Furthermore, the blue light only condition consistently outperformed caffeine when both congruent and incongruent distractions were presented. The visual reactions in the absence of a decision or distraction were also enhanced in the blue light only condition and this effect was most prominent in the blue-eyed participants. Overall, blue light and caffeine demonstrated distinct effects on aspects of psychomotor function and have the potential to positively influence a range of settings where cognitive function and alertness are important. Specifically, despite the widespread use of caffeine in competitive sporting environments, the possible impact of blue light has received no research attention.

## Introduction

Much like the ear has functions for hearing and balance, the human eye has a dual role in detecting light for a range of behavioural and physiological responses that are distinct from their role in vision [1]. These non-image forming light effects are known to modulate multiple endocrine, behavioural and physiological responses including melatonin suppression, heart rate elevation, enhancement of alertness, mood and performance, and stimulation of gene expression [2-9]. The non-visual alerting effects of light have been shown to be mediated, at least in part, by a melanopsin-dependent photoreceptive system which is specifically sensitive to blue light (wavelengths of 460-480 nm) [10,11].

The alerting response to blue light is effective in reducing sleepiness and enhancing cognitive performance, and specifically in tasks associated with concentration and cognition [12-14]. Indeed, the ability of short light wavelengths to modulate alertness via the suppression of melatonin is well established [15,16] with greater suppression noted in light eyed Caucasians [17]. However, the acute suppression of melatonin is not requisite for light to have an effect on measures of alertness at night [18] and short wavelength light has been demonstrated to elicit enhanced alertness and cognitive performance during daylight hours even when the contribution of melatonin suppression is indubitably negligible [19].

Caffeine is a methylxanthine well known to improve mood [20,21], vigilance and alertness [22,23] via acceleration of motor processes through central and/or peripheral mechanisms [24,25]. The beneficial psychomotor performance effects of caffeine can be regarded in some respects, as having similar effects to light of short wavelengths. However; day-time blue-enriched light has been reported to have a positive effect on subsequent sleep which contrasts with the negative effects on sleep that have been reported with caffeine administration [26]. This would indicate that the effects of blue light and caffeine, although comparable, are distinct under different circumstances.

The intention of the current study was to compare and contrast the physiological responses to blue light and caffeine, administered both separately and conjointly. Measures of cognitive function, reaction time and wakefulness were assessed and it was hypothesized that similarities would be observed with the administration of 240 mg of caffeine and a 1 h dose of ~40 lx blue light. Further, it was hypothesized that a combination of blue light exposure and caffeine ingestion would induce alerting and psychomotor effects greater than either intervention in isolation and that eye colour would influence the degree of the psychomotor and physiological responses to blue light.

## Methods

### Ethics Statement

The investigation was conducted according to the principles expressed in the Declaration of Helsinki. All participants provided informed written consent prior to participation with pre-approval obtained from the Umeå Regional Ethical Review Board (# 2012-318-31M).

### Participants

The study recruited 24 subjects (13 male, 11 female; 26 ± 4 y). Individuals were excluded if they had worked night shifts during the last 12 months or had travelled through more than one time zone during the last 2 months. All participants were non-smokers, low to moderate caffeine and alcohol consumers, and were not on contraindicatory medication. Colour blind subjects were not excluded as normal trichromatic vision is not necessary for light-mediated neuroendocrine regulation [27].

### Experimental Protocol

Participants reported to the Östersund test facility (63.1° N) on four separate test sessions within a one-month period during winter (February-March). All participants refrained from drinking caffeinated products or alcohol, and strenuous physical exercise on trial days. Prior to testing the participants were familiarized with a computer-based psychomotor vigilance test protocol (PVT) and eye colour was assessed visually. On four intervention days participants arrived at the test facility in the evening (17:00 to 18:00 h) after at least a two hour fast and completed the PVT (described below) that consisted of 20 trials of a visual and audio Go/No-Go test, an Eriksen Flanker test, and 5 trials of a visual reaction time task (all tasks are available online at www.cognitivefun.net). The PVT lasted approximately 10 minutes

After performing the PVT, the participants ingested a gelatine capsule containing either 240 mg of caffeine or a visually indistinguishable sugar placebo with a small glass of water. They then rated their level of alertness using the Karolinska Sleepiness Scale (KSS) before entering a separate room specifically set up to deliver either ~40 lx of blue light from a LED light source (Techlight® RGB, 3W, λmax = 470 nm) or a white light alternative (~100 lx) for 1 h. Thus there were four trial conditions administered in a random manner to avoid sequence effects: white light/placebo (PLA); white light/240 mg caffeine (CAF); blue light/placebo (BLU); blue light/240 mg caffeine (BCAF). Each trial was of equivalent duration and lasted 1 h during which the participants were consistently exposed to the light stimulus and were instructed to keep their eyes open while remaining comfortably seated and listening to relaxing music. Every 15 minutes a researcher entered the room to re-evaluate the level of alertness using the KSS and ensure subject compliance. At the end of the 1 h light exposure, the participants again completed the PVT.

#### Psychomotor Vigilance Test (PVT)

The first test of the PVT was a Go/No-Go task, which has previously been used to assess sustained attention [12,14]. The Go/No-Go Test involved the random presentation of 20 visual stimuli and then 20 audio stimuli and the participant was required to strike the space bar on the computer keyboard (Go), or inhibit that action (ignore the stimulus) when a non-cued stimuli was presented (No-Go). The fastest and average reaction times were recorded for analysis.

The Eriksen Flanker task is an executive function test used in cognitive psychology to assess psychomotor vigilance and the ability to suppress responses that are inappropriate in a particular context. In the task, a directional response was made to a central target stimulus by striking the left or right arrow on the computer keyboard. The target was randomly flanked by non-target stimuli which corresponded either to the same directional response as the target (*congruent* flankers) or to the opposite response (*incongruent* flankers). The fastest and average reaction times for both the congruent and incongruent presentations were recorded for analysis.

The visual reaction time task consisted of five presentations of a visual stimulus on the computer screen, where the participant was instructed to strike the space bar as quickly as possible upon presentation. The fastest reaction time was recorded for analysis. The Go/No-Go, Eriksen Flanker, and visual reaction time tasks were all performed on the same computer installed with the f.lux program (available online at www.stereopsis.com) to minimise extraneous blue light emission that has been demonstrated to influence psychomotor performance [14].

#### Karolinska Sleepiness Scale (KSS)

The KSS was used to subjectively assess alertness as this test is used for evaluating subjective sleepiness [28] and has been reported to be a “useful proxy” for electroencephalographic (EEG) activity [29]. The scale has anchor points which range from 1 (very alert) to 9 (very sleepy, an effort to stay awake, fighting sleep).

### Statistical Analyses

An analysis of variance with factors for caffeine dose and light treatment was utilized to determine differences in the means of the response variables resulting from the four treatments. Differences were interpreted in relation to the likelihood of exceeding the smallest worthwhile effect with individual change thresholds for each variable. Variables were log- or arsineroot-transformed when appropriate, to reduce bias arising from non-uniformity of error. Magnitudes of the standardized effects (Cohen Effect Size [ES]) were interpreted using thresholds of 0.2, 0.6, 1.2 and 2.0 for small, moderate, large, and very large respectively [30]. Standardized effects of between -0.19 and 0.19 were termed trivial. Quantitative chances of higher or lower differences were evaluated qualitatively as follows: <1%, almost certainly not; 1–5%, very unlikely; 5–25%, unlikely; 25–75%, possible; 75–95%, likely; 95–99%, very likely; >99%, almost certain. To make inferences about the large-sample value of an effect, the uncertainty in the effect was expressed as 90% confidence limits (CL). An effect was deemed unclear if the confidence interval overlapped the thresholds for both small positive and small negative effects. The significance level was set at p≤ 0.05.

## Results

**Figure 1:**
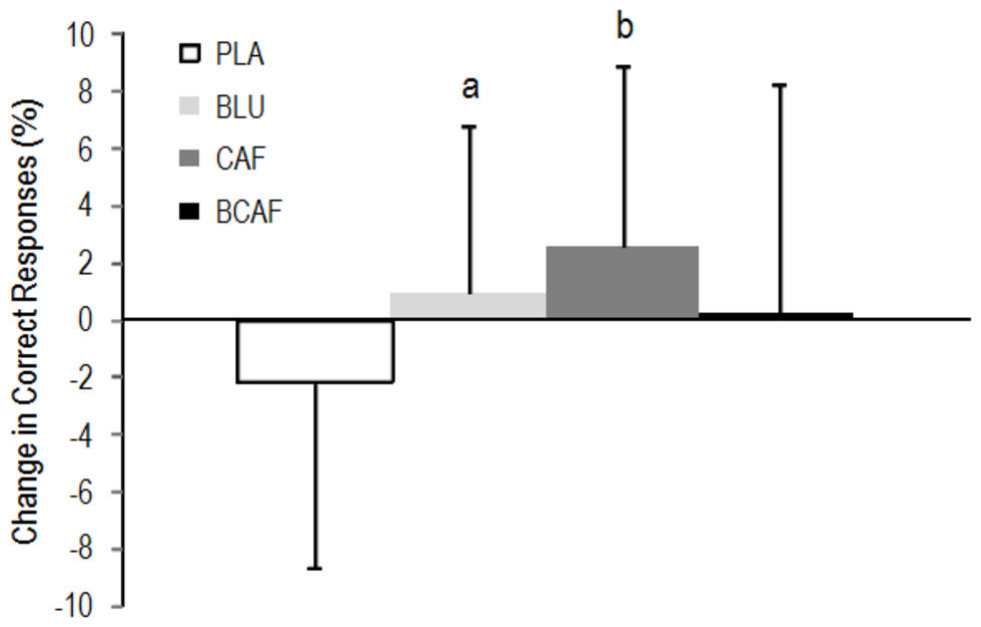
Change in the accuracy in the visual Go/No-Go psychomotor task. a: small difference from PLA, b: moderate difference from PLA. PLA: white light/placebo; CAF: white light/240 mg caffeine; BLU: blue light/placebo; BCAF: blue light/240 mg caffeine.

Twenty-one participants (13 male, 8 female) completed the entire experimental procedure with an average relative caffeine dose of 3.2 ± 0.5 mg·kg. The number of correct responses in the visual Go/No-Go task decreased in the PLA condition (Figure 1) with a clear difference between the positive effect of CAF (ES: 0.66) and BLU (ES: 0.50). No accuracy differences (% correct) were observed between the conditions in the audio Go/No-Go task. Post-treatment differences in the Go/No-Go task were observed between the average audio reaction times (BLU < CAF; ES: 0.49) and for the fastest visual reaction time (BCAF < PLA; ES: 0.43).

**Figure 2:**
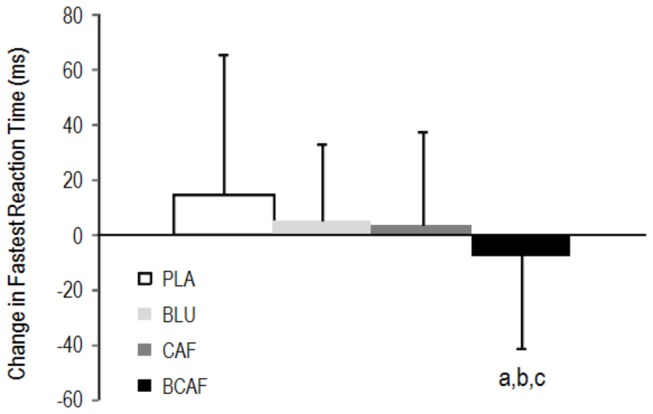
Change in performance of the visual Go/No-Go psychomotor task. a: small difference from BLU, b: small difference from CAF, c: moderate difference from PLA. PLA: white light/placebo; CAF: white light/240 mg caffeine; BLU: blue light/placebo; BCAF: blue light/240 mg caffeine.

The BCAF condition caused an improvement in the fastest visual Go/No-Go reaction time task (Figure 2) that was superior to PLA (-8.7 ± 7.1%; ES: 0.72), CAF (-4.4 ± 6.0%; ES: 0.44) and BLU (-5.3 ± 5.3%; ES: 0.45).

**Figure 3:**
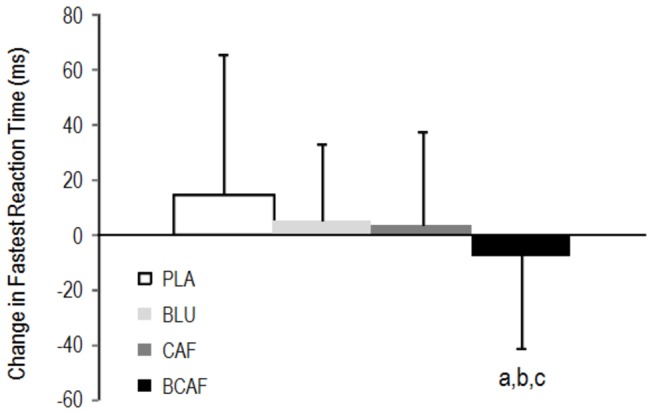
Change in the accuracy in incongruent presentations in the Eriksen flanker psychomotor task. a: large difference from CAF, b: large difference from BCAF, c: moderate difference from CAF, d: moderate difference from BCAF. PLA: white light/placebo; CAF: white light/240 mg caffeine; BLU: blue light/placebo; BCAF: blue light/240 mg caffeine.

**Figure 4:**
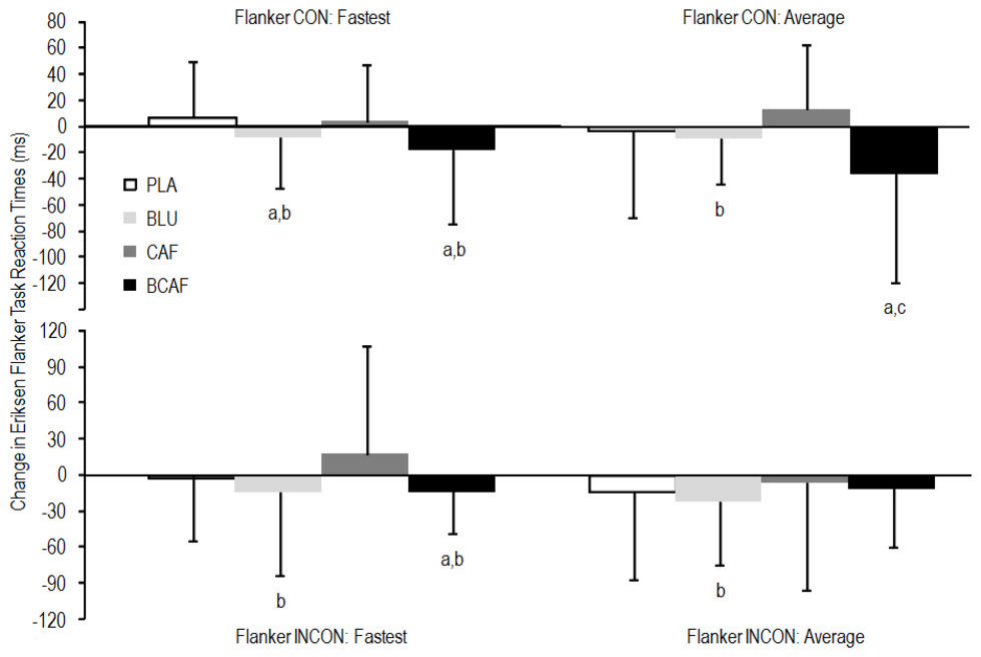
Change in performance of the Eriksen flanker psychomotor task. a: small difference from PLA, b: small difference from CAF, c: moderate difference from CAF. PLA: white light/placebo; CAF: white light/240 mg caffeine; BLU: blue light/placebo; BCAF: blue light/240 mg caffeine, CON: Congruent flanker presentations, INCON: Incongruent flanker presentations.

With respect to the Eriksen Flanker task, there was a significant difference between the congruent and incongruent reaction times (359 vs 382 ms; p = 0.0141). Caffeine ingestion had a large negative impact on the percentages of correct responses in the incongruent presentation (Figure 3). In the congruent presentations, the only observed difference in the change from pre- to post-intervention was a small accuracy improvement in the PLA condition compared to the BCAF condition (ES: 0.53). The change in performance (reaction time) in the Eriksen Flanker task is depicted in Figure 4. The blue light treatment consistently showed beneficial performance effects when compared to the CAF condition.

**Figure 5:**
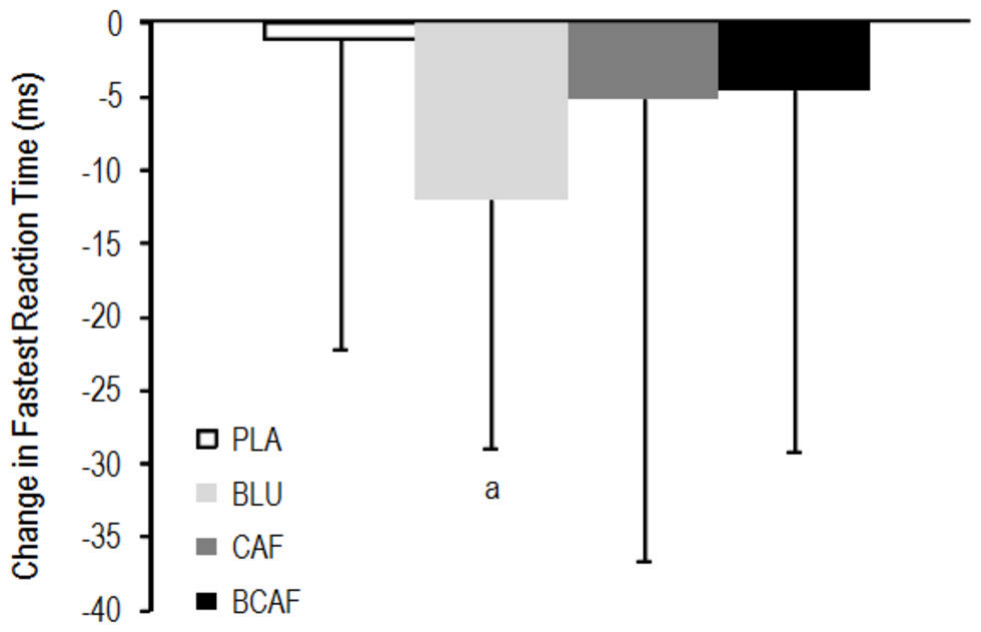
Change in performance of the visual reaction time task. a: small difference from PLA. PLA: white light/placebo; CAF: white light/240 mg caffeine; BLU: blue light/placebo; BCAF: blue light/240 mg caffeine.

Males had faster reaction times than females in the visual reaction time task (255 vs 274 ms; p = 0.0172). Post-treatment differences in reaction times were observed between the conditions: PLA > BLU (6.3 ± 5.2%; ES: 0.65), PLA > CAF (5.5 ± 5.6%; ES: 0.53) and BCAF > BLU (4.2 ± 4.6%; ES: 0.47). The changes in reaction times from pre- to post-intervention are presented in Figure 5.

Interestingly, the improved reaction times seen after the blue light treatment in Figure 5 (ES: 0.41; p = 0.0410) were driven by the blue-eyed participants (n = 14), with no difference in reaction time observed in the non-blue-eyed participants (n = 7; p = 0.3840). Similarly, within the BCAF treatment, there was a clear difference between the response of the blue-eyed and non-blue-eyed participants (5.8 ± 6.5%; ES: 0.50).

**Figure 6:**
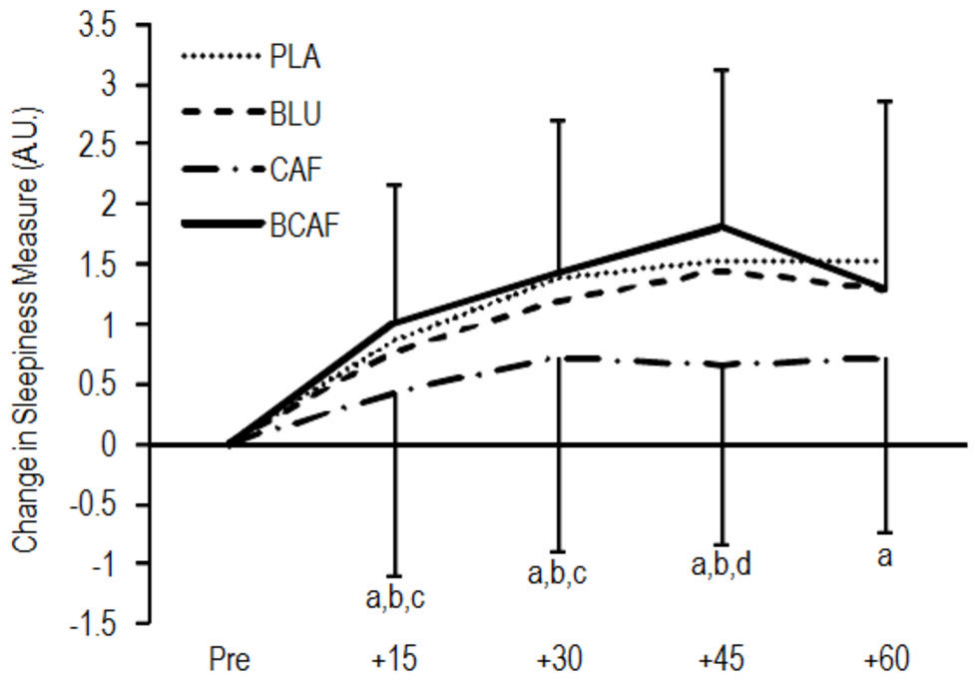
Change in Karolinska sleepiness scale ratings over time. a: small difference from PLA, b: small difference from BLU, c: small difference from BCAF, d: moderate difference from BCAF. PLA: white light/placebo; CAF: white light/240 mg caffeine; BLU: blue light/placebo; BCAF: blue light/240 mg caffeine, AU: Arbitrary units.

The mean pre-treatment subjective alertness levels were 4.1 ± 1.5 units. The changes in subjective measures of alertness resulting from the experimental conditions are presented in Figure 6. No relationships were observed between the post-treatment KSS or the change in KSS scores and any measures of cognitive function or psychomotor ability.

## Discussion

Here, we demonstrate for the first time that blue light and caffeine demonstrate distinct effects on aspects of psychomotor function, presumably via distinct mechanisms. Previously, a combination of bright white light and caffeine has been assessed as a countermeasure for impaired alertness and performance during extended sleep deprivation [31]. Interestingly, the effects of the blue light treatment in our visual reaction time task were mediated, in part, by the eye colour of the participants. Thus, we contend that the previously observed influence of eye pigmentation on wavelength-specific melatonin suppression [17] can be extended to psychomotor effects, and further that this effect is independent of ethnicity.

Both blue light exposure and caffeine ingestion improved accuracy in the visual Go/No-Go task; however, their combination did not result in enhancement in the number of correct responses (Figure 1). It has been proposed that the effects of caffeine follow the Yerkes-Dodson law in which it is postulated that the relationship between arousal and performance follows an inverted U-shape curve [24]. Thus, if acting upon similar brain architecture, it is possible to rationalize that the combined treatment of blue light and caffeine dose exceeded the optimal state of arousal and consequently resulted in impaired accuracy. However, the combination of blue light with caffeine did result in an improvement in the fastest reaction time in the visual Go/No-Go task in line with previous research using short wavelength light [12,14] which suggests an additive effect of the treatments (Figure 2).

As seen in previous research, the response times in the Eriksen Flanker task were slower for incongruent stimuli than for congruent stimuli [32]. Also similar to earlier research [12] we found no little or no difference between the blue light treatment and our placebo condition in the Eriksen Flanker task designed to assess executive function (Figures 3 and 4). Accuracy data did however demonstrate a clear *decrease* in accurate responses to incongruent presentations when caffeine was administered (Figure 3). It is possible that this decrease in performance is related to the anxiogenic properties of caffeine. While caffeine is known to improve alertness and can improve cognitive and motor function performance [24,31], studies have reported negative effects of caffeine consumption on hand steadiness [33] and anxiety [34]. While there was a small difference between the PLA and BLU treatments in the fastest reaction time in the congruent presentation, the blue light treatment consistently outperformed CAF in all tasks (Figure 4).

Our subjective sleepiness data derived from the Karolinska Sleepiness Scale demonstrated that the well known effect of caffeine on the participants’ perception of alertness occurred quite rapidly (Figure 6). However, we did not see a similar effect on subjective alertness in either the BLU or BCAF treatment groups. Previous researchers have reported decreases in both objective and subjective measures of sleepiness as a result of short wavelength light exposure [1,14], although non-significant effects of light treatment have also been observed [18]. It is worth noting then, that the alerting effects of the blue light treatment highlighted above occurred independently of subjective sleepiness which may have important ramifications given the known negative impact of caffeine on sleep latency and quality [35].

Although caffeine is known to improve alertness and can improve cognitive and motor function performance [24,31], as well as athletic skill execution [36], numerous studies report negative effects of caffeine consumption with impacts on hand steadiness [33], sleep latency and quality [35] and anxiety [34]. Caffeine administration (2 mg·kg) has also been associated with impaired recall in male subjects presented with a 12-item word list [37]. It has been suggested that many of the effects of caffeine constitute a return to normal function, or an amelioration of the effects of caffeine withdrawal, and thus does not improve psychomotor function above ‘normal’ levels [38]. However, a number of researchers have demonstrated that caffeine has psychoactive effects even in the absence of withdrawal [39-41]. It should also be noted that some of the effects of caffeine have been attributed to expectancy [42] and that, despite the double-blinded study design, we cannot discount the possibility that some participants were conscious of the physiological stimulatory actions of caffeine and adjusted their subsequent behaviours accordingly. Further, individual responses to caffeine are commonly reported in literature and it has been suggested that individual differences in sensitivity result from intrinsic differences in responsiveness to caffeine at sites of action in the brain, rather than from differences in absorption, distribution, or metabolism of the substance [43].

The light intensity in the current study of ~40 lx has been used previously to induce an alerting effect after a 1 h exposure [44]. The difference in the light intensity between the PLA and BLU treatments demonstrates that any alerting effects observed in the blue light treatment group were as a result of the wavelength of light and independent of light intensity. The current study utilised an exposure time of 1 h in order to match the duration of effect with caffeine which is absorbed and demonstrates physiological effects after 45-60 min when administered orally [24]. It is known however, that just 50 s of short-wave light exposure can cause detectable effects in the hypocampus and amygdala, brain areas associated with arousal [45], and thus can affect cognitive functions almost instantaneously. Further, the exposure time and intensity has been suggested to influence the nature of the response to light treatment, the persistence of the response, and the brain regions affected [11,45,46].

A dual-pathway has been hypothesized to explain the physiological and psychological outcomes of blue light exposure [47]. A limbic pathway that involves the amygdala is hypothesized to work in parallel with the circadian pathway (retinohypothalamic tract) to elicit the light-induced improvements in subjective alertness independent of melatonin suppression. MRI studies have shown that daytime exposure to blue light, when compared with green, almost instantaneously enhances brain responses to a memory task in several cortical, thalamic, and brainstem areas [48]. The direct projection to the amygdala from melanopsin-expressing photosensitive ganglion cells is believed to explain the rapid limbic responses to light with subsequent modulation of brain activity in cortical areas upon prolonged exposure [46].

The alerting effects of short-wavelength light have led to the suggestion that light can be used as a non-pharmacological countermeasure in a range of occupational settings [1]. However, the potential for light therapy to impact athletic performance has been overlooked. Here we show improvements in reaction time as a result of blue light exposure and such improvements may have athletic implications, especially given recent research demonstrating a link between reaction time and sprint performance [49]. Further, since reaction time is an important component of agility [50], blue light has the potential to enhance athletic performance as numerous sports are performed indoors with artificial lighting conditions and / or at night time.

Here, we combined for the first time the known alerting effects of blue light exposure with the widely used stimulant caffeine and demonstrated some divergent effects of the two treatments. In instances where a rapid decision in response to a visual stimulus was required, additive positive effects were observed; however caffeine demonstrated negative effects on tasks of executive function with an inability to suppress inappropriate responses in the context of our Eriksen Flanker task. Indeed, blue light consistently improved executive psychomotor function when compared to oral caffeine administration. Blue light also demonstrated positive effects on visual reaction time, an effect that was more pronounced in blue-eyed participants. This alerting effect could have benefits in a variety of occupational and contexts including competitive sporting environs.
